# *Helicobacter pylori*: Routes of Infection, Antimicrobial Resistance, and Alternative Therapies as a Means to Develop Infection Control

**DOI:** 10.3390/diseases12120311

**Published:** 2024-12-03

**Authors:** Ayman Elbehiry, Adil Abalkhail, Nuha Anajirih, Fahad Alkhamisi, Mohammed Aldamegh, Abdullah Alramzi, Riyad AlShaqi, Naif Alotaibi, Abdullah Aljuaid, Hilal Alzahrani, Feras Alzaben, Mohammed Rawway, Mai Ibrahem, Moustafa H. Abdelsalam, Nermin I. Rizk, Mohamed E. A. Mostafa, Moneef Rohail Alfaqir, Husam M. Edrees, Mubarak Alqahtani

**Affiliations:** 1 Department of Public Health, College of Applied Medical Sciences, Qassim University, P.O. Box 6666, Buraydah 51452, Saudi Arabia; 2 Medical Emergency Services Department, Faculty of Health Sciences, Umm Al-Qura University, Al-Qunfudah P.O. Box 1109, Saudi Arabia; 3 Department of Preventive Medicine, King Fahad Armed Hospital, Jeddah 23311, Saudi Arabia; 4 Pathology and Laboratory Medicine Department, Armed Forces Hospital-Jubail, Jubail 31951, Saudi Arabia; 5 Medical Radiology Department, Armed Forces Hospital-Jubail, Jubail 31951, Saudi Arabia; 6 Biomedical Engineer, Armed Forces Medical Services, Riyadh 12426, Saudi Arabia; 7 Medical Hospital Administration Department, Armed Forces Hospital-Jubail, Jubail 31951, Saudi Arabia; 8 Medical Hospital Administration Department, Armed Forces Hospitals in Al Kharj, AL Kharj 16278, Saudi Arabia; 9 Physical Medicine and Rehabilitation Department, Armed Forces Center for Health Rehabilitation, Taif 21944, Saudi Arabia; 10 Department of Food Service, King Fahad Armed Forces Hospital, Jeddah 23311, Saudi Arabia; 11 Biology Department, College of Science, Jouf University, Sakaka 42421, Saudi Arabia; 12 Botany and Microbiology Department, Faculty of Science, Al-Azhar University, Assiut 71524, Egypt; 13 Department of Public Health, College of Applied Medical Science, King Khalid University, Abha 61421, Saudi Arabia; 14 Department of Physiology, Faculty of Medicine, University of Tabuk, Tabuk 74191, Saudi Arabia; 15 Department of Anatomy, Faculty of Medicine, University of Tabuk, Tabuk 74191, Saudi Arabia; 16 Department of Radiology, King Fahd Armed Forces Hospital, Jeddah 23311, Saudi Arabia

**Keywords:** *Helicobacter pylori*, disease transmission, antibiotic resistance, alternative therapies, infection control

## Abstract

*Helicobacter pylori* (*H. pylori*) is a Gram-negative, spiral-shaped bacterium that colonizes the gastric epithelium and is associated with a range of gastrointestinal disorders, exhibiting a global prevalence of approximately 50%. Despite the availability of treatment options, *H. pylori* frequently reemerges and demonstrates increasing antibiotic resistance, which diminishes the efficacy of conventional therapies. Consequently, it is imperative to explore non-antibiotic treatment alternatives to mitigate the inappropriate use of antibiotics. This review examines *H. pylori* infection, encompassing transmission pathways, treatment modalities, antibiotic resistance, and eradication strategies. Additionally, it discusses alternative therapeutic approaches such as probiotics, anti-biofilm agents, phytotherapy, phototherapy, phage therapy, lactoferrin therapy, and vaccine development. These strategies aim to reduce antimicrobial resistance and enhance treatment outcomes for *H. pylori* infections. While alternative therapies can maintain low bacterial levels, they do not achieve complete eradication of *H. pylori*. These therapies are designed to bolster the immune response, minimize side effects, and provide gastroprotective benefits, rendering them suitable for adjunctive use alongside conventional treatments. Probiotics may serve as adjunctive therapy for *H. pylori*; however, their effectiveness as a monotherapy is limited. Photodynamic and phage therapies exhibit potential in targeting *H. pylori* infections, including those caused by drug-resistant strains, without the use of antibiotics. The development of a reliable vaccine is also critical for the eradication of *H. pylori*. This review identifies candidate antigens such as *VacA*, *CagA*, and *HspA*, along with various vaccine formulations, including vector-based and subunit vaccines. Some vaccines have demonstrated efficacy in clinical trials, while others have shown robust immune protection in preclinical studies. Nevertheless, each of the aforementioned alternative therapies requires thorough preclinical and clinical evaluation to ascertain their efficacy, side effects, cost-effectiveness, and patient compliance.

## 1. Introduction

*Helicobacter pylori* (*H. pylori*) is a spiral-shaped, flagellated, Gram-negative, microaerophilic bacterium that thrives under specific growth conditions [1,2,3]. Initially isolated in 1983 from patients diagnosed with antral gastritis [4]. *H. pylori* has been implicated in various gastrointestinal disorders, including gastritis, peptic ulcers, and certain malignancies [5]. In 2017, the International Agency for Research on Cancer classified *H. pylori* as a Class I carcinogen [6]. The bacterium possesses several virulence factors that enhance its pathogenic potential, including resistance to acidic environments and antibiotics [7,8,9,10]. Notably, factors such as Cytotoxin-Associated Gene A (*cagA*) and Vacuolating Cytotoxin A (*vacA*) have been associated with the development of gastric carcinoma [7,11,12]. Research suggests that the flagella of *H. pylori* facilitate its penetration into the submucosa of the stomach [13,14,15], while the urease enzyme contributes to its survival in acidic conditions [16,17,18].

*H. pylori* infection can occur early in life via oral–oral or oral–fecal routes, with natural elimination rare without antimicrobials [19]. Approximately 4.5 billion people are infected worldwide, contributing to 9% of cancer-related deaths [20]. Infection rates are 15–25% in wealthy countries and 75–90% in underdeveloped countries [21,22]. The infection is more prevalent in impoverished regions compared to industrialized areas. Factors contributing to this gap include health issues, family finances, ethnicity, and the number of individuals affected [23]. Exposure to *H. pylori* increases the likelihood of infection due to prolonged tobacco use, insufficient vitamin intake, high salt consumption, and living conditions that alter stomach pH [24].

*H. pylori* is primarily transmitted through oral–oral and fecal–oral routes [24]. The bacterium is present in the saliva, feces, and vomit of infected individuals, facilitating transmission [25]. It often spreads within families in developing countries, especially between infected mothers and their children [26], though partner transmission is uncertain [27,28]. The exact route to the human stomach is unclear, but environmental contamination likely plays a role [24]. Poor hygiene can contaminate treated water [24], and studies indicate that water may transfer *H. pylori* from feces to the mouth. Infection is more common in children using external water sources or consuming raw vegetables irrigated with untreated wastewater [29,30]. Food can also become contaminated in unsanitary conditions, and milk, along with vegetables and meat, has been studied for its role in transmission [31].

The crisis of antimicrobial resistance against pathogenic bacteria is considered an urgent matter worldwide [32,33,34,35,36,37,38,39,40,41]. A key factor in the failure of *H. pylori* eradication programs is antibiotic resistance [42]. Proton pump inhibitors (PPIs), combined with two classes of antimicrobials and bismuth, are the standard treatment for *H. pylori* infection, but eradication rates have declined due to drug resistance [20]. The World Health Organization (WHO) has reported a troubling increase in antimicrobial resistance, with some antibiotics, like metronidazole and clarithromycin, showing resistance levels of 15% or more. [20]. Resistance to clarithromycin rose from 15.6% in the early 2000s to over 40% by 2020. Metronidazole resistance increased from 58% in the early 2000s to 78% in 2020, as indicated by Garvey et al. [43]. Meanwhile, Savoldi et al. [20] reported that standard triple treatment is less than 80% effective in eradicating *H. pylori*.

Medical authorities advise discontinuing triple antibiotic therapy if antimicrobial resistance exceeds 15%, as per the Maastricht IV/Florentine Consensus Report [44]. In these cases, quadruple therapy, which includes two antimicrobial agents, PPIs, and bismuth salts, may be used. However, since quadruple therapy still contains antibiotics, individuals resistant to these should avoid it. Due to a shortage of bismuth salts, initiating quadruple therapy with bismuth is impractical in many countries where its use is restricted [45,46]. Furthermore, Poonyam et al. [47], have shown that antimicrobial agents can cause digestive issues, including diarrhea, eating disorders, vomiting, and abdominal discomfort. There are significant safety concerns regarding antimicrobial treatment in older adults, children, and pregnant women, making it inadvisable for these populations [48].

Complementary therapies are gaining popularity for managing *H. pylori* infections. Using probiotics and herbal medicines alongside antibiotics can mitigate antibiotic side effects and reduce resistant organisms [49]. Lactoferrin (LF) inhibits bacterial growth by depriving bacteria of iron and enhancing membrane permeability [50,51]. LF may also help treat *H. pylori* infections and gastric ulcers due to its anti-inflammatory effects [52,53]. Currently, phage therapy has shown promise in treating various illnesses, including chronic conditions [54]. However, its use against *H. pylori* may be delayed [55], because the understanding of *H. pylori* phage biology is still developing. *H. pylori* vaccination is rapidly advancing [1], with about ten antigen types and nearly ten adjuvant types identified to enhance the immune response [56]. Various delivery technologies have been developed to improve antigen presentation, and several clinical studies are underway, offering new hope for eradicating *H. pylori* infection [57,58,59,60]. There is a high demand for alternative drugs to control *H. pylori* infections.

Therefore, this review examines *H. pylori* infection, focusing on transmission pathways, treatment modalities, antibiotic resistance, and eradication strategies, which include tailored therapy and potassium-competitive acid blockers. Additionally, it discusses alternative therapeutic approaches such as probiotics, anti-biofilm agents, phytotherapy, phototherapy, phage therapy, lactoferrin therapy, and vaccine development. The objective of these strategies is to mitigate antimicrobial resistance and enhance treatment outcomes for *H. pylori* infections.

## 2. Methodological Methods

**Figure 1:**
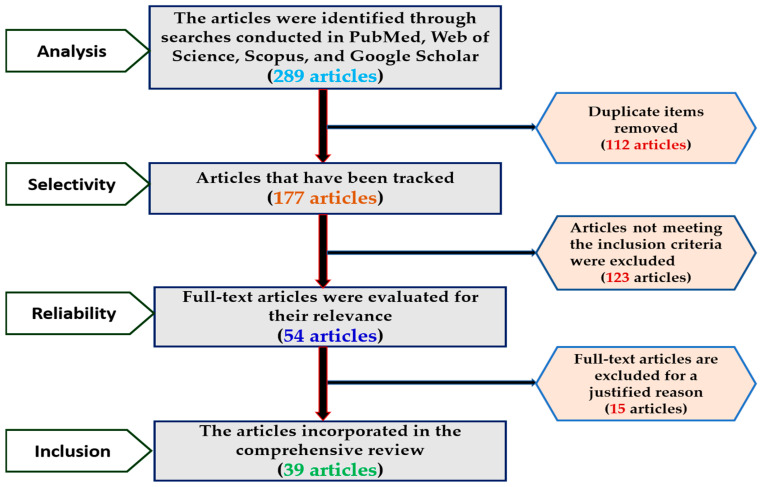
Flow chart explaining the review process for manuscripts that have been screened.

The review process was executed following a flowchart, as illustrated in Figure 1, which delineates the steps for evaluating manuscripts that have successfully undergone the screening process. This review involved a comprehensive literature analysis aimed at collecting information on *H. pylori*, with particular emphasis on transmission pathways, treatment failures, antimicrobial resistance, and alternative therapeutic strategies. The inclusion criteria encompassed original research articles, review papers, and clinical trials that addressed antibiotic resistance, conventional treatment modalities, and alternative interventions for *H. pylori*. Key topics explored included the potential applications of alternative medicine, instances of treatment failure, and vectors of infection. To ensure the relevance and timeliness of the research, only English-language publications from 1983 to 2024 were considered. The analysis specifically excluded non-research materials such as editorials and commentaries, non-English publications, duplicate studies, and research that did not pertain to *H. pylori* transmission mechanisms, antibiotic resistance, alternative therapies, or treatment failures. Searches were performed across databases including PubMed, Web of Science, Scopus, and Google Scholar, utilizing keywords such as “*H. pylori*”, “transmission routes”, “antibiotic resistance”, “alternative therapy”, “treatment failure”, “probiotics”, “phage therapy”, and “vaccine”. The quality of the included studies was evaluated based on predetermined criteria.

## 3. The Transmission Patterns of *Helicobacter pylori*

The mechanisms underlying the transmission of *H. pylori* remain inadequately understood. Consequently, there is an urgent need to develop a more comprehensive understanding of the pathways through which *H. pylori* disseminates into the gastric environment, thereby enhancing human resistance to such infections. Given that *H. pylori* appears to have a restricted host range, it is posited that its primary host is the human gastrointestinal tract, where infection can occur [24,61]. New infections are believed to arise from environmental exposure or direct interpersonal contact. Generally, there are three principal modes through which humans may become infected with *H. pylori*.

### 3.1. Human-to-Human Transmission

*H. pylori* infections are primarily transmitted through person-to-person contact, with two main modes: vertical and horizontal. Horizontal transmission occurs through contact with non-family members or environmental contamination. Vertical transmission is the transfer of an infectious agent from one generation to the next within the same family [62]. Many studies have investigated the link between *H. pylori* infection and familial susceptibility. The majority of the studies [26,63] indicate that *H. pylori* infections frequently cluster in families. *H. pylori* can spread within families through direct transmission [1,64]. Factors such as close relationships, genetic predisposition, shared socioeconomic conditions, and common sources of infection contribute to this spread [65,66,67,68]. Yang et al. [14] highlight that transmission is especially common in households with frequent mother–child interactions. Research suggests that *H. pylori* infections may cluster in families, and a study by Ding et al. [69] found that children can acquire the infection from infected parents.

Childhood infection risk is significantly affected by environmental factors and family dynamics. Children in larger families with more siblings are more likely to contract *H. pylori* infections [70], with mothers and grandparents typically serving as primary caregivers. Young children can contract *H. pylori* through oral and fecal pathways by consuming food chewed by a caregiver, being kissed on the mouth, or if the caregiver fails to wash their hands after using the bathroom [71]. Thus, children with relatives who have had *H. pylori* infections are also at risk [72]. A 2013 study in Japan found that grandmothers significantly contribute to *H. pylori* transmission across generations [73]. Goodman and Correa [74] indicated that older family members are more likely to transmit *H. pylori* to younger siblings, especially those close in age. Fialho et al. [75] found that *H. pylori* can be transmitted from younger to older relatives, indicating possible sibling transmission. Patel et al. [76] discovered that children in economically disadvantaged schools in Edinburgh had a significantly higher prevalence of *H. pylori* infection than those in other regions, even when controlling for other risk factors. Although *H. pylori* transmission likelihood decreases with age, Brenner et al. [77] found that couples can still contract the infection. In a study of 670 couples in Germany, the infection prevalence was 34.9% in women and 14.5% in uninfected husbands.

According to a study conducted in the medical field, 82.4% of gastrointestinal endoscopy specialists had *H. pylori* infections in their stomachs. The infection rate among gastrointestinal healthcare professionals is 16.8%, whereas it can reach 70% among dental professionals, according to Kehre et al. [78]. As a result, work-related variables are important conduits through which potential *H. pylori* infection can spread. The spread of *H. pylori*-contaminated saliva can also occur through the use of shared utensils by individuals who are infected with this bacterium and healthy individuals who are not. In a study of 328 adult Chinese immigrants living in Melbourne, Australia, Chow et al. [79] examined the prevalence of *H. pylori* infection and found significant associations with utensil transmission in both male and female infectors. Although some evidence suggests that *H. pylori* can be transmitted by utensils, a study published by Leung et al. [80] found that the presence of *H. pylori* was only 3.7% in infected cases and 10% in salivary-infected cases, indicating low odds of transmission by utensils. A summary of the data shows that *H. pylori* most commonly affects children and adolescents; however, feeding utensils pose a small risk of infection.

### 3.2. Animals to Human’s Transmission

*Helicobacter* species can infect humans and domesticated animals, such as dogs, cats, pigs, and birds, as well as wild animals like monkeys [81,82,83,84]. A *Helicobacter* bacterium similar to those in animals with gastritis has also been found in humans with gastritis [85]. A large segment of the global population lives near domestic animals, especially dogs and cats, highlighting the importance of these findings [86]. While some animals, such as sheep and dogs, can temporarily carry *H. pylori*, the impact on humans is still uncertain. Factors that may increase the risk of *H. pylori* infection include childhood exposure to unpasteurized milk, raw vegetable consumption, and contact with pets like dogs and cats. *H. pylori* can be transmitted zoonotically, mainly through indirect means [87]. This infection is a key transmission pathway from animals to humans, especially in developing countries. Duan et al.’s work, published in 2023, notes that *H. pylori* infects both humans and animals [88]. Papież et al. [89] found higher *H. pylori* infection rates among sheep ranchers and their families in the Tatra Mountains of Poland (97.6% and 86%, respectively) compared to farmers without sheep (65.1%) [90]. Several studies have indicated that *H. pylori* can be detected in milk [91,92], poultry slaughterhouses [93], and other fresh foods.

### 3.3. Transmission Through Water and Food

Water is vital for human survival, posing a risk of contact with *H. pylori*-contaminated sources [94,95,96]. *H. pylori* can survive in various water types, including cold, salty, distilled, and tap water, due to its ability to modify peptidoglycans in its cell walls. Contaminated water is a primary vector for *H. pylori* transmission, often linked to fecal matter [97]. Klein et al. [98], found that children from high-income families using municipal water are twelve times more likely to contract *H. pylori* than those using well water, indicating greater contamination in city supplies. *H. pylori* infections can also result from contaminated food, with fecal contamination of drinking water being a primary transmission route through streams, rivers, lakes, soil, and groundwater. Studies show high levels of *H. pylori* in Iranian water bottles and an increasing infection rate among those using non-municipal water for toilets [99,100]. Individuals at higher risk may consume water from polluted sources. Preventing *H. pylori* spread requires better dietary management and rigorous water testing.

**Figure 2:**
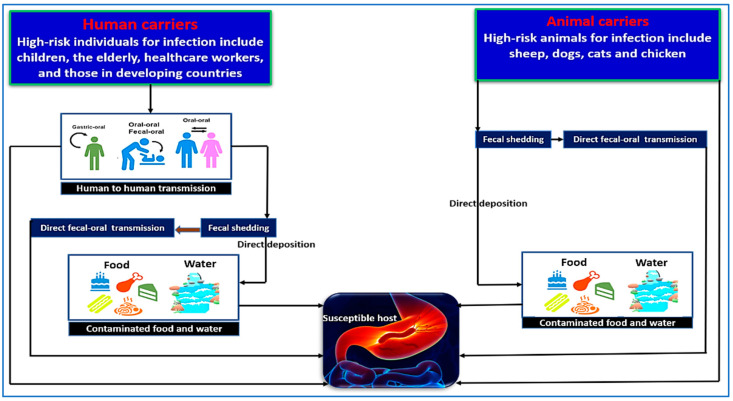
The pathways through which *H. pylori* is transmitted. Person-to-person transmission occurs among children, the elderly, healthcare workers, and individuals living in developing countries. The bacterium can spread through oral–oral, fecal–oral, or gastric–oral routes, as well as through fecal shedding that contaminates food or water sources. Oral–oral transmission may occur when sharing food utensils or between mothers and their newborns. Additionally, *H. pylori* can be transmitted to animals such as sheep, dogs, cats, and chickens through fecal shedding or direct fecal–oral contact. Food and water sources contaminated with *H. pylori* can also directly transmit the bacteria to susceptible individuals.

Contaminated water with *H. pylori* poses a serious risk, as it can taint fruits and vegetables, resulting in foodborne illnesses [101]. Hemmatinezhad et al. found that 28% of 50 fruit salads tested positive for *H. pylori* through molecular analysis [91]. The risk of infection can be reduced through thorough cleaning and avoiding contaminated water sources [91]. Hamada et al. [93] examined 90 samples of chicken meat, gizzards, and liver from a semi-automated slaughterhouse in Sadat City, Egypt, finding that seven samples (7.78%) tested positive for *H. pylori*. Similarly, Mashak et al. [102] tested 600 raw meat samples from Iranian slaughterhouses for *H. pylori*. Mutton contamination was 13.07%, while goat mutton was 11.53%. A study by Shaaban et al. [92] found *H. pylori* in 5 of 13 milk samples from farm animals. Figure 2 shows the main routes of *H. pylori* infection in humans, with food and water as potential sources. The risk of transmission rises with close contact between infected individuals and livestock. Regular handwashing and sanitizing are crucial for health and safety. Regularly examining water sources is crucial for identifying *H. pylori* infection origins and reducing transmission risk. Thoroughly clean fruits and vegetables before consumption and limit fresh meat and dairy intake.

## 4. *H. pylori* Infection: Standard Therapy, Antimicrobial Resistance, and Failure of Treatment

When choosing the optimal therapy, it is essential to consider regional antibiotic resistance and antimicrobial susceptibility testing results [103,104]. In some countries, a recommended treatment plan may include mixed therapy, administering multiple medications simultaneously for two weeks or more [105,106,107]. Triple therapy, combining amoxicillin, clarithromycin, and a PPI like omeprazole, has historically been the first-line treatment for *H. pylori* [13,107,108]. However, a 2016 study by Thung et al. [109] revealed significant antibiotic resistance, leading to the recommendation of second-line treatments. In the U.S. and Europe, quadruple therapy with metronidazole, tetracycline, omeprazole, and bismuth is now advised [47,110]. Clarithromycin’s minimal effect on stomach pH and effective mucosal diffusion make it essential in combination therapy for *H. pylori* infections [111]. The global prevalence of *H. pylori* and its related diseases is largely due to clarithromycin’s reduced effectiveness and recurrence in countries with poor healthcare infrastructure [112]. Therefore, the use of antimicrobial agents for *H. pylori* infections should be limited.

**Figure 3:**
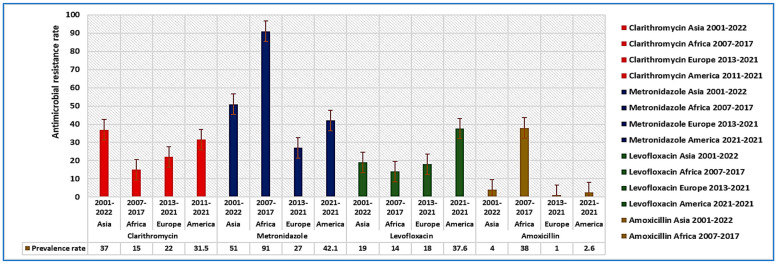
The prevalence rates of multidrug-resistant *H. pylori* across various regions, including Asia (2001–2022), Africa (2007–2017), Europe (2013–2021), and America (2011–2022). The resistance rates to clarithromycin were found to be 37% in Asia, 15% in Africa, 22% in Europe, and 31.5% in America. For metronidazole, the resistance rates were 51% in Asia, 91% in Africa, 27% in Europe, and 42.1% in America. The resistance rates to levofloxacin were reported as 19% in Asia, 14% in Africa, 18% in Europe, and 37.6% in America. Lastly, amoxicillin resistance rates were recorded at 4% in Asia, 38% in Africa, 1% in Europe, and 2.6% in America.Resistance mechanisms primarily stem from mutations that alter pharmacological targets. Drug-resistant genotypes are linked to mutations affecting membrane permeability, biofilm formation, and efflux pumps [120,128]. Amoxicillin resistance mainly arises from changes in membrane permeability and mutations in the penicillin-binding protein gene [120]. *H. pylori* strains often resist clarithromycin due to point mutations in *23S rRNA*. A study at Peking University utilized next-generation sequencing to identify genetic factors enhancing resistance to levofloxacin and clarithromycin [129]. Key mutation sites for clarithromycin resistance include peptidyl transferases in the *23S rRNA*, with A2143G and A2142G being the most common. Mutations in the DNA gyrase (*gyrA*) gene (N87K, D91N, D91G) were linked to levofloxacin resistance [129]. Reduced drug influx due to structural changes in lipopolysaccharide (LPS) membranes also contributes to resistance. Mutations in the *rfaF* (LPS heptosyltransferase II) gene lead to deep, coarse LPS drug absorption [130] and causing slight resistance to chloramphenicol, along with cross-resistance to amoxicillin, tetracycline, and clarithromycin [131]. Increased expression of *tolC* homolog genes (*hefA*) in patients with gastrointestinal disorders in Iran [132] was linked to efflux pump induction, as shown by real-time PCR in metronidazole and clarithromycin-resistant bacteria. The multidrug-resistant phenotype was found in 9.5% of cases. A genome-wide analysis identified prevalent mutations, including A2143G in *23S rRNA* (63.1%) and alterations in the *rdxA* gene (85.5%).

A meta-analysis by Boyanova et al. [113], found that *H. pylori* strains in Bulgaria had 30% resistance to clarithromycin and 42% to metronidazole. Savoldi et al. [20], reported that clarithromycin resistance in Europe was about 18%, compared to 33% in the western Pacific and 34% in the Mediterranean [114]. Antimicrobial resistance rates differ significantly between industrialized and developing nations [20,109,115,116,117,118,119,120]. Resistance to metronidazole and clarithromycin is notably higher than for other antibiotics [119]. In China, clarithromycin resistance has increased from 14.8% to 52.6% [109]. Over the past century, *H. pylori* has increasingly shown resistance to antibiotics like clarithromycin, amoxicillin, and metronidazole [121,122]. Figure 3 illustrates studies from Asia, Africa, Europe, and America that examined resistance rates for clarithromycin [123,124,125,126], metronidazole [66,125,126,127], levofloxacin [124,125,126,127], and amoxicillin [124,125,126,127] from 2001 to 2022, 2007 to 2017, 2013 to 2021, and 2011 to 2021, respectively.

**Figure 4:**
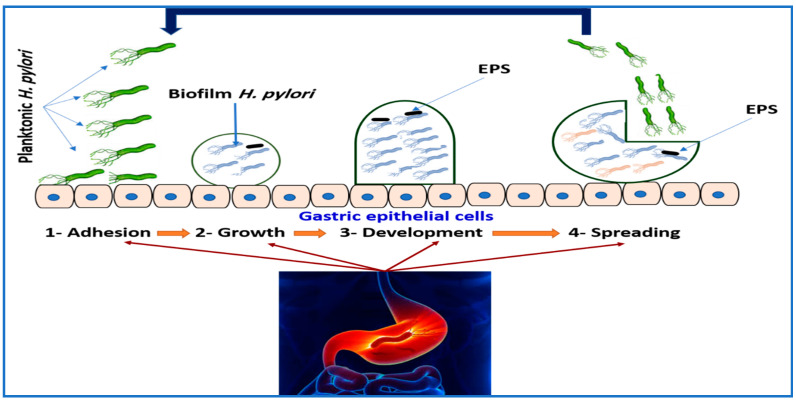
An overview of the biofilm formation process: (1) Attachment stage of biofilm formation involves reversible and irreversible processes. Reversible attachment occurs when planktonic cells adhere to surfaces via chemical interactions, aided by virulence factors like adhesins and pili, triggering biofilm formation and increasing microbial susceptibility to antimicrobials. (2) Growth (irreversible attachment) leads to microbial proliferation and colony establishment, enhancing adherence through transcriptional changes. This phase promotes substrate exchange, metabolic product distribution, and byproduct excretion. *H. pylori* secrete EPS, which lower biofilm cell susceptibility to host defenses and antimicrobials. (3) Development features an increasing extracellular matrix around microcolonies, driven by EPS production and quorum-sensing communication, both vital for resistance. Mature biofilms have high EPS content and interstitial spaces for nutrient, water, and planktonic cell movement. (4) Spreading occurs when detachment due to nutrient depletion prompts cells to seek new surfaces through erosion and sloughing.

Hou et al. [133] found that *H. pylori’s* resistance to antimicrobial agents is the main factor in biofilm development (Figure 4). Extracellular polymeric substances (EPSs) coat microbial surfaces and, due to their negative charge, hinder the penetration of antimicrobial agents, making microbes up to a thousand times more resistant to antibiotics than planktonic bacteria [133,134,135]. Administering antibiotics to *H. pylori* during biofilm formation is ineffective, as the antibiotics cannot penetrate the biofilm, resulting in unsuccessful therapy. Biofilms also protect *H. pylori* from the immune system, increasing antibiotic resistance [133]. Patients requiring repeated therapy for *H. pylori* often need a second treatment long after the first. *H. pylori* can switch from a spiral to a spherical shape, entering a viable but nonculturable state (VBNC), which cannot be cultivated [3,136,137]. Microbes can endure stressful conditions, such as sub-inhibitory drug dosages or unfavorable environments, without damage [97]. Chaput et al. [138] noted a significant alteration in the peptidoglycan of spherical *H. pylori* cells, allowing them to evade immune recognition while still stimulating IL-8 production in the stomach epithelium. This enables *H. pylori* to avoid or modulate the host immune response in a viable but non-culturable (VBNC) state, facilitating long-term survival in the stomach.

Wang and Wang [139] developed a population of spherical *H. pylori* by treating these cells with a sublethal dose of antimicrobial agents. Researchers confirmed the pathogenicity of spherical *H. pylori* cells by analyzing sequences from various strains. The study found a complete *cagA* gene in the bacteria, with about 99% similarity to the original sequence of vegetative forms. These results indicate that phenotypic changes are crucial for maintaining *H. pylori’s* “health” and survival throughout its life cycle [140]. The polymer substances and coccoid formation of *H. pylori*, along with the efflux pump on its membrane, contribute to drug resistance [133]. The efflux pump expels antimicrobial agents, reducing their intracellular concentrations [128,141,142]. In *H. pylori*, efflux pumps are key players in multidrug resistance [143]. Biofilms exposed to clarithromycin show significantly higher resistance than planktonic organisms, with increased expression of efflux pump genes [144,145]. Microorganisms producing biofilms are more likely to express the efflux pump genes Hp605, Hp971, Hp1327, Hp1489, Hp118, and Hp1174 than those forming planktonic structures [144,146]. Efflux pumps and biofilms work together to enhance drug resistance.

## 5. Alternative Therapies

**Figure 5:**
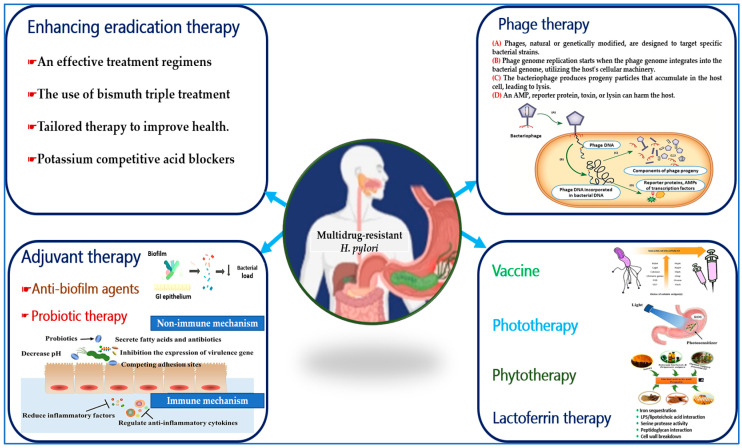
A range of alternative therapeutic approaches has been developed to combat the antimicrobial resistance exhibited by *H. pylori*. Probiotic therapy employs non-immune mechanisms to counteract *H. pylori* by competing for attachment sites, inhibiting the expression of virulence genes, and inducing cell death. Additionally, it reduces inflammatory mediators and regulates anti-inflammatory cytokines through immune mechanisms. Photodynamic therapy effectively eradicates *H. pylori* by generating ROS using a light source in conjunction with a photosensitizer. Phage therapy involves the production of progeny particles from bacteriophages that lyse host cells. The development of vaccines utilizing virulence antigens is crucial for reducing colonization and eradicating *H. pylori*. Phytotherapy can inhibit urease activity, prevent bacterial adhesion, and enhance membrane permeation against *H. pylori* infection. Lactoferrin therapy sequesters iron, interacts with lipopolysaccharides and lipoteichoic acids, modulates serine protease activity, and engages with peptidoglycan, ultimately leading to the collapse of the cellular wall.

The rise of antimicrobial resistance in *H. pylori* has complicated treatment. If eradication fails, multiple rounds of different antimicrobial combinations may be needed. Physicians are now tasked with finding effective alternatives to declining traditional therapies, which often have higher pill burdens and side effects [147]. Expert guidelines have shifted, now favoring quadruple therapy with bismuth as the initial treatment over the previous triple therapy with clarithromycin [148]. With limited options for antibiotic-resistant strains, innovative treatments, including non-antibiotic approaches, are urgently needed to address this issue (Figure 5).

### 5.1. Enhancing Eradication Therapy

*H. pylori* eradication regimens, developed by gastrointestinal specialists, aim to eliminate this bacterium. However, global success rates have declined, and antimicrobial resistance has increased [149]. A more targeted approach using specific antibiotics is needed to improve outcomes [105]. Customizing therapy strategies to regional sensitivity profiles is essential for addressing varying antimicrobial resistance trends [105,150]. However, the lack of reliable statistics on antibiotic-resistant bacteria in local communities hinders decision-makers from selecting effective empirical eradication strategies. Regions with high metronidazole- and clarithromycin-resistant *H. pylori* are advised to use bismuth triple therapy for infection elimination [151,152,153]. Adding bismuth to certain protocols and extending treatment to two weeks can boost eradication rates by up to 30% in resistant strains [154]. Botija et al. [155] conducted an evaluation of the efficacy of colloidal bismuth subcitrate (CBS) therapy in eradicating *H. pylori* among patients aged 5 to 8 years. They utilized data from a national pediatric registry comprising 682 patients. Among these patients, 38 (5.6%) received CBS treatment, with 50% of this group having experienced prior unsuccessful eradication attempts. A follow-up assessment of 32 patients revealed an eradication rate of 93.8% for those treated with CBS, compared to an 86.7% eradication rate for patients who did not receive CBS treatment (*p* < 0.05). Recent meta-analyses show that first-line regimens with bismuth have higher eradication rates than those without [156]. Nijevitch and colleagues [157] assessed the effectiveness of a triple therapy of nifuratel, amoxicillin, and bismuth for pediatric *H. pylori* gastritis. After endoscopy for dyspeptic symptoms, 73 children aged 9 to 14 received a 10-day course of treatment. *H. pylori* was eradicated in 63 participants (86%; 95% CI: 76.6–93.2). There were no withdrawals due to side effects, and no severe adverse reactions occurred.

The rising rates of resistance and multidrug-resistant *H. pylori* underscore the need for better detection of antimicrobial susceptibility and treatment [158]. Tailored therapy effectively increases eradication rates while reducing unnecessary antibiotic use [159]. This approach allows for drug selection based on susceptibility to antimicrobial agents determined by drug composition. In 2022, Nyssen et al. [160] conducted a meta-analysis examining the empirical and susceptibility-guided treatment approaches for *H. pylori*, which encompassed 54 studies involving a total of 6705 patients in the empirical treatment cohort and 7895 patients in the susceptibility-guided cohort. The results indicated that the eradication rates of *H. pylori* were significantly higher in the susceptibility-guided group, achieving an 86% success rate, in contrast to a 76% success rate observed in the empirical treatment group. Gingold-Belfer et al. [161] performed a meta-analysis of 16 randomized controlled trials to compare susceptibility-guided therapy and empirical therapy for *H. pylori* infection. The study involved 2451 patients receiving empirical treatment and 2374 receiving susceptibility-guided therapy. The findings revealed no significant difference in effectiveness, with a relative risk (RR) of 1.02 (95% confidence interval: 0.92–1.13; *p* = 0.759; I^2^ = 80%). Although empirical regimens effectively eradicate *H. pylori*, the advantages of tailored therapy may not be clear. Challenges include the absence of *H. pylori* cultures and antibiotic susceptibility testing. More research is needed to promote the widespread use of tailored treatments for *H. pylori* elimination.

While proton pump inhibitors with triple therapy are effective for acid reflux, there is growing interest in acid-suppressing medications [162]. The dual and triple use of potassium-competitive acid blockers (P-CABs) is an innovative and effective method for eradicating *H. pylori* [163]. Acid suppression is vital in *H. pylori* treatment, as a higher stomach pH fosters bacterial growth and increases susceptibility to antibiotics [164]. Lowering stomach pH stabilizes medications like clarithromycin and amoxicillin, which require acid suppression to prevent excessive acidity [165]. Vonoprazan, a P-CAB, is more effective than proton pump inhibitors and provides longer acid suppression [166]. Elazazi et al. [167] conducted a clinical investigation to evaluate the efficacy of *H. pylori* eradication protocols using Vonoprazan compared to proton pump inhibitors. The study involved 232 treatment-naïve participants, split into two groups: Arm 1 (58 patients) received clarithromycin, amoxicillin, and vonoprazan, while Arm 2 (58 patients) received clarithromycin, amoxicillin, and esomeprazole. Group II included treatment-experienced patients in Arm 3 (intervention) and Arm 4 (comparator), each with 58 participants. Arm 3 received levofloxacin, vonoprazan, nitazoxanide, and doxycycline, while Arm 4 received levofloxacin, esomeprazole, nitazoxanide, and doxycycline. All participants followed their treatment regimens for 14 days, with *H. pylori* eradication assessed four weeks later. Arm 3 had a 50% eradication rate, compared to 43.1% in Arm 4. Arm 1 achieved 58.6%, and Arm 2 recorded 50%. Regimens containing P-CABs were acceptable, with few adverse events. This therapy is beneficial with amoxicillin or amoxicillin plus clarithromycin for eradicating *H. pylori*. Japan and several other countries have approved a triple-treatment regimen including vonoprazan for *H. pylori* eradication [168,169].

### 5.2. Adjuvant Therapies (Probiotics and Anti-Biofilm Agents)

Adjuvant medicines aim to enhance antimicrobial therapy by combating antibiotic resistance or modifying the host response [147]. Probiotics, defined by the WHO and FAO, are live organisms that confer health benefits when administered in adequate amounts [170]. Beneficial probiotics in clinical settings include *Lactobacillus*, *Bifidobacterium*, *Bacillus*, *Streptococcus*, and *Escherichia coli*, which produce lactic acid [171]. Once inside the human body, these microorganisms produce antimicrobial compounds like lactic acid, hydrogen peroxide, and bacteriocins that kill bacteria. Lactic acid inhibits the urease activity of *H. pylori* [172], and reactive oxygen species (ROS) from probiotics can damage bacterial cell walls and membranes [173]. A recent meta-analysis found that most probiotics in triple therapy improved outcomes with standard eradication therapy [174,175]. Mohtasham et al. [174] conducted a double-blind, randomized controlled trial with 450 participants to assess probiotics as an adjuvant to quadruple therapy for *H. pylori* eradication. Participants received a 14-day treatment of bismuth subcitrate, pantoprazole, amoxicillin, and clarithromycin, along with either a probiotic (*Lactobacillus ruteri*, 100 mg) or a placebo. After eight weeks, the urea breath test showed slightly higher eradication rates in the probiotic group (per-protocol: 80.1% vs. 75.2%; intention-to-treat: 78.7% vs. 72%), though not statistically significant. However, only 69.7% of the probiotic group reported side effects, compared to 98.6% in the placebo group (*p* < 0.001), and they experienced fewer gastrointestinal adverse effects, except for constipation (*p* < 0.001).

The application of probiotic therapy has shown greater effectiveness in eradicating *H. pylori* infections [170]. Probiotics have the ability to reduce *H. pylori* colonization by strengthening the stomach’s mucosal barrier and competing with pathogenic bacteria for adherence [176]. This could potentially help manage diseases linked to *H. pylori*. Numerous studies have suggested that probiotics have minimal adverse effects on patients’ digestive systems, increasing the chances of compliance [170,177]. Probiotics may inhibit *H. pylori* colonization, maintain the gastric mucosal barrier, and reduce gastric inflammation. They can also modulate the host’s immune response to infection [147]. Probiotic supplements can help restore intestinal microbiota balance disrupted by antibiotics [178,179]. Yuan et al. [180] studied the effects of *H. pylori* eradication and probiotics on gastric microbiota in young adults. The study included 95 *H. pylori*-positive participants and 56 negative controls, aged 19 to 30, assigned to probiotics monotherapy, probiotics-supplemented quadruple therapy, or quadruple therapy alone. Gastric mucosal samples were collected before treatment and two months later for 16S rRNA gene sequencing. Two months post-eradication, the gastric microbial composition differed significantly from *H. pylori*-negative participants, with decreased alpha diversity in gastric juice and increased diversity in gastric mucosa. Probiotic-assisted eradication improved microbial diversity compared to quadruple therapy, increasing *Bifidobacterium* and *Lactobacillus* while reducing harmful bacteria like *Fusobacterium* and *Campylobacter*. Probiotic monotherapy had limited effects on *H. pylori* and beneficial bacteria but significantly altered gastric microbiota diversity, leading to an increase in potentially harmful bacteria post-treatment.

Probiotics exert diverse molecular effects based on their characteristics and chemical composition, leading to beneficial outcomes through various mechanisms [181]. They interact directly with gastrointestinal cells, releasing bioactive compounds that act as signaling molecules in the interactions among intestinal immune cells, gut microbiota, and epithelial cells [182,183]. Key molecular effectors include proteins, low molecular weight peptides, amino acids, bacterial DNA, and short-chain fatty acids (SHFAs) [184]. Probiotic antigens can penetrate the intestinal barrier and trigger immune responses. They enhance intestinal barrier selectivity by increasing mucin, immunoglobulin A (IgA), and defensins, while also boosting the synthesis of vitamins, minerals, SCFAs, and growth regulators [176,185]. Probiotics further promote antiangiogenic factors, cytokines like interleukin-2 (IL-2) and interleukin-12 (IL-12), and antioxidants, which help lower intestinal pH. Lastly, they regulate apoptosis and cell differentiation by inhibiting harmful pathways such as tyrosine kinase [170]. Further research is needed to fully understand the mechanisms and functions of probiotics in eradicating *H. pylori*.

Another treatment option targets bacterial biofilms with anti-biofilm agents, primarily derived from natural products such as phytochemicals, biosurfactants, antimicrobial peptides, and microbial enzymes [186,187]. Probiotics and quorum-sensing inhibitors also effectively inhibit biofilm growth [188,189]. Almost all natural substances can act as antibacterial agents against *H. pylori* biofilms [133]. Natural products show anti-biofilm and antibacterial properties against *H. pylori* strains resistant to multiple antimicrobial agents [190,191,192]. N-acetylcysteine (NAC), a dietary supplement with anti-inflammatory and antioxidant effects, effectively treats *H. pylori* infections [193,194] and can reduce bacterial load while enhancing eradication rates [195,196,197]. NAC treatment before antibiotics improves *H. pylori* clearance, as shown in a clinical trial [198]. However, the exact mechanism of NAC’s effects on biofilm disruption and antimicrobial resistance in *H. pylori* is still unknown. Moreover, combining antimicrobial agents with rhamnolipid, a glycolipid biosurfactant that disrupts biofilms and may reduce bacterial adhesion in vitro, effectively inhibits biofilm development [199,200].

## 6. Other Developing Therapies

### 6.1. Lactoferrin Therapy

Lactoferrin (LF) is an iron-binding protein in the transferrin family [201] with antiviral, antibacterial, antioxidant, and anti-inflammatory properties [51]. LF levels rise significantly during *H. pylori* infections, correlating with gastric mucosal inflammation [51]. It is crucial for maintaining iron balance and aids in iron absorption in the intestinal tract [202,203]. LF inhibits bacterial growth by depriving them of essential iron and increasing membrane permeability [50]. Yamazaki et al. [204] conducted a study on the antibacterial effects of lactoferrin and Lactoferricin^®^ against *H. pylori* in vitro. Bovine Lactoferricin^®^ was found to be effective at concentrations above 5.0 mg/L, while human and bovine lactoferrins had minimum bactericidal concentrations of 1.25 to 2.50 mg/mL. Both compounds showed dose-dependent effects during exponential growth. Bovine Lactoferricin^®^ exhibited modest activity in brucella broth but had rapid effects in 1% Bacto-peptone medium at concentrations of 0.1 to 1.0 mg/mL. Iron-saturated lactoferrin did not inhibit growth, but bovine Lactoferricin^®^ reduced *H. pylori* urease activity. These findings suggest that *H. pylori* is susceptible to both compounds, with the effectiveness of lactoferrin depending on the bacterium’s iron status and growth phase, unlike Lactoferricin^®^.

Wada et al. [205] studied the effects of bovine lactoferrin (bLF) on germ-free BALB/c mice infected with *H. pylori*. After oral inoculation, the mice received daily bLF for two to four weeks. Results showed that 10 mg of bLF increased *H. pylori* presence tenfold while significantly reducing its attachment to the gastric epithelium. Consequently, serum antibody titers for *H. pylori* became undetectable, indicating a weakened immune response. These findings suggest that bLF has a direct antibacterial effect and can detach *H. pylori* from the stomach epithelium. Ciccaglione et al. [206] found that combining bovine LF with levofloxacin, amoxicillin, and a proton pump inhibitor provided an additional 21% therapeutic effect in patients from areas with high antibiotic resistance. Other studies have shown that bovine LF inhibits *H. pylori* growth at pH 6, both in vivo [207] and in vitro [208].

Yuan et al. [53] examined the efficacy of goat-derived transgenic recombinant human LF against *H. pylori* in vitro and in vivo. Their in vitro findings showed that recombinant LF reduced the virulence factors *cagA* and *vacA* and inhibited *H. pylori* development. Lu et al. [209] also studied the effects of *H. pylori* infection on host LF levels using animal models. The study revealed that *H. pylori*-infected stomachs had LF levels 9.3 times higher than healthy stomachs. More recent research by Imoto et al. [51] found that bovine LF inhibits *H. pylori* growth in vitro at concentrations of 25.2 to 50.0 mg/mL. LF is often combined with antibiotics to treat *H. pylori* infections effectively [44] and has been shown to improve treatment success rates. In the future, LF combined with antibiotics may replace traditional triple therapy as a more effective option.

### 6.2. Herbal Therapy (Phytotherapy)

Herbal therapy, or phytotherapy, involves using plants and their extracts for medicinal purposes [210,211]. Various plant parts—leaves, stems, flowers, roots, and seeds—are used to create raw or processed herbal products [212]. Health regulations classify herbs as nutritional additives, allowing them to be sold without prior safety or efficacy evaluations [213]. The effectiveness of herbal therapies relies mainly on empirical evidence due to limited scientific research [214]. Controlled clinical trials are vital for assessing the efficacy of herbal medicines and improving their quality and safety [215]. Li et al. [216] examined the effects of Banxia Xiexin Decoction (BXXXT), a traditional Chinese medicine prescription, on drug-resistant *H. pylori*-induced gastritis in mice using both in vivo and in vitro methods. The aqueous extract (BXXXT) was prepared through water decoction. In vitro tests demonstrated BXXXT’s inhibitory effects on *H. pylori*, while an acute gastritis model was established in vivo. Treated mice were assessed for *H. pylori* colonization, gastric mucosal repair, inflammation, and apoptosis. The minimum inhibitory concentration (MIC) of BXXXT was found to be 256–512 μg/mL, with a dosage of 28 mg/kg proving more effective than standard triple therapy. The extract consisted of at least 11 compounds, including berberine and quercetin, which exhibited synergistic effects and enhanced immune function in CD3+ and CD4+ T cells. Certain plants and fruits contain compounds such as flavonoids, terpenoids, and alkaloids that may effectively treat *H. pylori* infections [217,218].

Fahmy et al. [219] found that flavonoids from Erythrina speciosa (Fabaceae) had the lowest minimum inhibitory concentration (MIC) against *H. pylori*. Zardast et al. [220] reported that raw garlic significantly reduced *H. pylori* growth in the stomach mucosa within 72 h. The ethyl acetate extract from this plant showed the highest antimicrobial activity, with an MIC of 62.5 µg/mL. Ayoub et al. [221] evaluated the essential oils and methanol extracts of *Pimenta racemosa* (*P. racemosa*) leaves and stems for their inhibitory activities against *H. pylori*, both in vitro and in silico. The essential oil from the stems showed significant antibacterial activity with a MIC of 3.9 µg/mL, comparable to clarithromycin’s MIC of 1.95 µg/mL. In silico studies suggested that compounds such as decanal, eugenol, terpineol, delta-cadinene, and amyl vinyl may inhibit *H. pylori* urease, indicated by strong binding affinity scores. These results highlight the therapeutic potential of *P. racemosa*, particularly in its stems, which are often considered agro-industrial waste. Shmuely et al. [222] noted that plant extracts inhibit urease, prevent adhesion, and permeate membranes to combat *H. pylori*. Fahmy and his colleagues [219] reported significant antimicrobial activity in plant extracts at MICs below 100 µg/mL, supporting the use of plants for treating *H. pylori* infections. These agents have proven effective in eliminating *H. pylori* and preventing related gastrointestinal disorders.

Herbal medicine offers numerous advantages, including widespread availability, affordability, and a significant presence among consumers who perceive it as a safer alternative to synthetic pharmaceuticals, particularly in regions with a longstanding tradition of herbal use [223]. The application of natural products may pose fewer risks compared to conventional treatments that often involve multiple antibiotics, although it is important to acknowledge that herbal remedies can also have side effects [224,225]. Research indicates that the integration of conventional therapy with ethnomedicine results in higher eradication rates and a reduction in adverse effects [226]. Furthermore, combination therapy has proven effective in alleviating symptoms of gastritis associated with Helicobacter pylori through a holistic approach [227]. Additionally, herbal therapy may mitigate antibiotic resistance due to its multitarget effects [227]. Patients who are unable to tolerate high doses of antibiotics may be particularly well-suited for herbal therapy.

### 6.3. Photodynamic Therapy

Photodynamic therapy (PDT) is a proposed method for eliminating harmful bacteria [228]. It generates ROS through the oxidation of biomolecules when a photosensitizer (PS) is exposed to laser light [229,230]. Unlike traditional antibiotics, PDT poses no risk of drug resistance, making it a promising alternative [231,232,233]. However, its application for treating *H. pylori* is still in early development, necessitating a PS that specifically targets *H. pylori* to protect normal cells from phototoxicity [234,235]. *H. pylori* produces sialic acid binding adhesin (*SabA*), which binds specifically to 2,3-linked sialic acid on sialyl-dimeric Lewis X antigens in the gastric epithelium. This binding promotes strong adhesion and colonization of the gastric mucosa [236,237]. The presence of 2,3-linked sialic acids in 3′-sialyl lactose (3SL) suggests it may effectively target *H. pylori*, as human cells lack 3SL receptors, making it highly selective for this bacterium [228,235,238].

The fundamental principle underlying PDT involves the generation of high ROS through the interaction of a PS, molecular oxygen, and visible light at an appropriate wavelength [239]. This process leads to the oxidation of various cellular components, resulting in rapid cell inactivation [240]. Numerous studies have identified potential targets for ROS generated by PDT within biofilms, particularly the EPS matrix [241], which comprises proteins [242], lipids [243], DNA [244], and extracellular polysaccharides [245]. Damage to proteins and DNA induced by PDT significantly diminishes the metabolic activity of the biofilm and may lead to structural disruption [246]. Phototherapy can effectively eliminate bacterial biofilms in the stomach, providing a therapeutic benefit against antibiotic-resistant bacteria [247]. Qiao et al. [248] studied the antibacterial effects of phototherapy on multidrug-resistant *H. pylori* using a near-infrared photosensitizer, T780T-Gu, created by combining guanidinium (Gu) with T780T. The results showed that T780T-Gu has synergistic effects in photothermal and photodynamic treatments against biofilms and MDR strains of *H. pylori*, potentially enhanced by structural deficits and reduced metabolism. Im et al. [228] developed a photomedicine called multiple 3SL-conjugated poly-L-lysine-based photomedicine (p3SLP) for targeted PDT against *H. pylori*. In C57BL/6 mice, oral administration of p3SLP followed by laser irradiation effectively inactivated *H. pylori* by targeting *sabA* on the bacteria’s membrane, without harming host cells. p3SLP shows potential as an endoscopic antibacterial PDT method for treating *H. pylori*.

### 6.4. Phage Therapy

Phage therapy, which uses bacteriophages to treat bacterial infections, has gained attention due to advancements in genetic engineering, metagenomics, high-throughput genome sequencing, and biotechnology [249,250,251]. Bacteriophages infect and destroy bacteria by attaching to specific receptors, introducing their genetic material, multiplying, and causing the bacterial cell to rupture, releasing more phages in the lytic cycle [252,253]. The destruction of bacterial cells helps maintain host cell health by eliminating pathogenic bacteria. Engineered phages can enhance their ability to target specific bacteria. Phage therapy may treat antibiotic-resistant infections in diverse patient populations and reduce antibiotic use in livestock [251].

Interest in phage therapy for *H. pylori* infections has grown [232]. This method employs bacteriophages to target and eliminate *H. pylori* [254]. Ferreira et al. [255] isolated the novel podovirus prophage HPy1R using *H. pylori* strains using UV radiation. It has a genomic length of 31,162 base pairs and encodes 36 predicted proteins, including 17 structural proteins. Phage particles remained stable at 37 °C and pH 3–11 for 24 h. In an in vitro stomach digestion model, only a slight reduction occurred during the gastric phase, indicating adaptation. This phage also reduced *H. pylori* levels for up to 24 h post-infection at multiplicities of infection of 0.01, 0.1, and 1 microaerophilic condition, suggesting its potential for phage therapy in the absence of exclusively lytic phages. Cuomo et al. [256] studied the effectiveness of *H. pylori*-specific lytic phage (*H. pylori* φ) alone and with lactoferrin adsorbed on hydroxyapatite (LF-HA) nanoparticles (*H. pylori* φ + LF-HA) in preventing *H. pylori* infection. The bacteria were obtained from human stomach biopsies and cultured in brain heart infusion (BHI) broth with 10% horse serum at 37 °C and 5% CO_2_ for phage isolation. The study found that LF-HA significantly enhances *H. pylori* φ activity, indicating that phages complexed with LF can selectively eliminate *H. pylori* without harming host cells, making it a promising therapeutic option. The *H. pylori* φ φ + LF-HA combination showed potential efficacy when administered at the onset of infection, but the minimum effective doses were not established.

A study examined the effects of lactoferrin on hydroxyapatite nanoparticles combined with a lytic phage, showing improved antibacterial effects in human gastric cancer cells [257]. Nonetheless, knowledge about phage-*H. pylori* interactions in the stomach microenvironment are still lacking. The limited availability of sequenced phage genomes restricts our understanding of *H. pylori* phages, including their pathogenicity, antimicrobial resistance genes, and toxins [232]. *H. pylori* phages lack endolysins [258], proteins that dissolve bacterial cell walls, and could serve as alternative treatments in phage therapy. Endolysins are host-specific, with no known bacterial resistance [259]. However, the protective outer layer of Gram-negative pathogens like *H. pylori* complicates treatment. Lysins can penetrate outer membranes when combined with mild acids or engineering techniques [260,261]. Despite the potential of phage therapy, more research is needed before it can be widely implemented for *H. pylori*.

### 6.5. Vaccination Against H. pylori: Potential Uses

An effective *H. pylori* vaccine could transform infection control and reduce future antibiotic use. While few candidates have shown promise in generating a protective immune response [56,262,263], several are under evaluation. The stomach’s acidic pH and the continuous renewal of mucosa allow *H. pylori* to evade the immune system. Even after eradication, patients may not remain protected [1]. A vaccine could prevent or reduce the frequency and severity of stomach infections [264]. To improve the effectiveness of preventive or therapeutic vaccinations, it is crucial to select appropriate adjuvants and immunogenic bacterial antigens [265]. Antigens such as Cytotoxin-associated gene A (*CagA*), vacuolating cytotoxin A (*VacA*), blood group antigen-binding adhesin (*BabA*), *H. pylori* adhesin A (*HpaA*), neutrophil-activating protein (*NapA*), outer inflammatory protein A (*OipA*), gamma-glutamyl transpeptidase (*GGT*), heat shock protein A (*HspA*), outer membrane proteins (*Omp*), and flagellar cap protein (*FliD*) have been linked to vaccinations [266]. Vaccines targeting four virulence proteins (FVPE) [267] and the multi-epitope vaccine (CTB-UE) [268] contain adjuvants and antigens expressed on CD4+ and CD8+ cells. Cholera toxin and *E. coli* enterotoxin are used as mucosal adjuvants to boost the immunogenicity of whole-cell and subunit vaccines. Furthermore, intramuscular *H. pylori* subunit vaccines with aluminum hydroxide adjuvants and oral live vector vaccines expressing *H. pylori* antigens are recommended for long-lasting protection [269].

In 2017, Guo and colleagues [270] developed the multivalent epitope-based vaccine CFAdE, using antigenic fragments from four *H. pylori* adhesins: *ure*, *Lpp20*, *HpaA*, and *cagL*. They assessed its specificity, immunogenicity, and ability to generate neutralizing antibodies in BALB/c mice, as well as its therapeutic efficacy and protective immune responses in *H. pylori*-infected Mongolian gerbils. CFAdE induces high levels of specific antibodies against urease, *Lpp20*, *HpaA*, and *cagL*. Oral vaccination with CFAdE and polysaccharide adjuvant (PA) significantly reduces *H. pylori* colonization compared to *ure* and PA immunization, with protection linked to IgG, sIgA antibodies, and antigen-specific CD4+ T cells. A multivalent epitope-based vaccine targeting multiple adhesins in *H. pylori* is more effective than a urease-targeting single epitope vaccine, offering a promising treatment for *H. pylori* infection. Adding a polysaccharide adjuvant to the multivalent vaccine dramatically reduced *H. pylori* levels in mice compared to the monovalent vaccine group [268]. As a result, multivalent vaccinations are becoming more popular. The vaccine developed by Guo and his colleagues targets *H. pylori* using bacterial attachment molecules, including urease, lipoprotein (*Lpp20*), *H. pylori* adhesins (*HpaA*), and *CagL*. Testing in experimental models showed increased antibody production against adhesion molecules in vaccinated mice [270]. Reports indicate that the deactivated *H. pylori* whole-cell vaccine enhances gastrointestinal immunity and reduces *H. pylori* severity [57].

In 2023, Katsande et al. [271] modified *Bacillus subtilis* spores to display *H. pylori* antigens, urease subunit A (*ureA*), and subunit B (*ureB*). They evaluated immunity and colonization in mice challenged with *H. pylori* after oral administration of these spores. Vaccination with *ureA* or *ureB*-expressing spores induced antigen-specific mucosal responses (fecal *sIgA*), seroconversion, and hyperimmunization, reducing *H. pylori* colonization by up to 1 log. This study highlights the potential of *Bacillus spores* for mucosal immunization against *H. pylori*, given their thermal stability and probiotic properties. Zeng et al. [272] conducted a Phase 3 clinical study in China to evaluate a three-dose oral recombinant *H. pylori* vaccine in healthy children aged six to fifteen. Participants without prior *H. pylori* infection were randomly assigned to receive the vaccine or a placebo, with the primary outcome being the incidence of infection within one year (ClinicalTrials.gov: NCT02302170). From 2 December 2004 to 19 March 2005, 4464 individuals were assigned to the vaccination (*n* = 2232) or placebo group (*n* = 2232), with 4403 (99%) completing the regimen. In the first year, 64 infections were reported: 14 in the vaccination group and 50 in the placebo group, resulting in a vaccine effectiveness of 71.8% (95% CI: 48.2–85.6). Adverse reactions occurred in 157 individuals (7%) in the vaccination group and 161 (7%) in the placebo group, with major events in five (<1%) and seven (<1%) individuals, none linked to the vaccine. While vaccination could help prevent *H. pylori* infections globally, no vaccine candidates have yet proven clinically relevant [232,273].

A comprehensive summary of various therapeutic studies aimed at eradicating *H. pylori* is presented in Table 1 below. This table includes detailed information regarding each potential therapy, encompassing the type of study (preclinical, clinical, in vitro, etc.) along with the results and outcomes associated with each investigation.

## 7. Conclusions

*H. pylori* infection poses a significant global health challenge, with gastric cancer as a common complication. Rising antibiotic resistance has led to interest in alternative treatments. This review clarifies transmission pathways, treatment failure, antimicrobial resistance, and emerging therapies. *H. pylori* is primarily transmitted through saliva and contaminated food or water. Developing countries are especially susceptible due to poor water treatment and hygiene. The infection spreads mainly among family members, particularly affecting children. Triple therapy with amoxicillin, clarithromycin, and a PPI like omeprazole has been the first-line treatment for *H. pylori*. Due to multidrug resistance, quadruple therapy with metronidazole, tetracycline, omeprazole, and bismuth is now recommended as a second-line option. Clarithromycin, metronidazole, levofloxacin, and amoxicillin are often linked to *H. pylori* drug resistance in developing countries due to altered membrane permeability, biofilm formation, and efflux pump activity. Alternative therapies, such as adjuvant therapy (probiotics and antibiofilm agents), phage therapy, phototherapy, phytotherapy, lactoferrin therapy, and vaccine development, are essential for treating *H. pylori* infection. Probiotics fight *H. pylori* by competing for attachment sites, inducing cell death, and regulating inflammatory cytokines. Phytotherapy inhibits urease activity and improves membrane permeability, though it is still in early development. Both probiotics and herbal therapies are effective second-line treatments due to their safety and lack of resistance. Phage therapy uses bacteriophages to lyse host cells. Photodynamic therapy generates ROS to eliminate *H. pylori*. Lactoferrin therapy sequesters iron and disrupts bacterial cell walls, making it a safe alternative. The development of vaccines targeting virulence antigens, such as *cagA* and *vacA*, is crucial for reducing *H. pylori* colonization and enhancing eradication strategies. However, further clinical evidence is needed to validate their practical implementation. Although vaccines, probiotics, and phages offer promising therapeutic alternatives, additional research is necessary to clarify the underlying mechanisms and assess their efficacy through rigorous clinical trials.

## Figures and Tables

**Table 1:** Summary of therapeutic studies contributing to the eradication of multidrug-resistant *H. pylori* infection.

Therapy	Type of Study	Study Description	Outcomes and Endpoints	References
Triple therapy plus colloidal bismuth subcitrate (CBS) therapy	Clinical	The study included children aged 5 to 18 with *H. pylori* infection identified by endoscopy in the Spanish Registry. It analyzed patients who received CBS treatment between 2020 and 2023, with 38 patients (5.6%) treated out of 682 registered.	CBS therapy has an eradication rate of 93.8%. The eradication rate for patients not receiving CBS therapy is 86.7%. In a subgroup of six patients on quadruple therapy with CBS, who were dual resistant to metronidazole and clarithromycin, the eradication rate was 100%.	[155]
	Clinical	Seventy-three pediatric outpatients (48 males, 25 females; ages 9–14) diagnosed with *H. pylori*-associated chronic gastritis and dyspeptic symptoms participated in the study. They underwent endoscopic evaluation and received a 10-day treatment of bismuth subcitrate (8 mg/kg/day), nifuratel (30 mg/kg/day), and amoxicillin (50 mg/kg/day), given four times daily. *H. pylori* infection status was evaluated before and 4 to 6 weeks after treatment using modified Giemsa staining.	The study involved 73 children (48 males and 25 females) aged 9 to 14 years. *H. pylori* was successfully eradicated in 63 patients, resulting in an efficacy rate of 86%. The 95% confidence interval for the efficacy rate was between 76.6 and 93.2. There were no serious adverse reactions or withdrawals reported during the study. The combination therapy used was nifuratel, bismuth subcitrate, and amoxicillin, which proved to be effective and well-tolerated.	[157]
Tailored therapy	Clinical	A meta-analysis assessed empirical and susceptibility-guided treatment approaches for *H. pylori*, involving 54 studies with 6705 patients in the empirical cohort and 7895 in the susceptibility-guided cohort.	Susceptibility-guided group achieved an 86% eradication rate for *H. pylori*. This rate is significantly higher than the 76% eradication rate in the empirical treatment group.	[160]
	Clinical	This meta-analysis reviewed 16 randomized controlled trials comparing susceptibility-guided therapy and empirical therapy for *H. pylori* infection, involving 2451 patients on empirical treatment and 2374 on susceptibility-guided therapy.	No significant difference in effectiveness was found. Relative risk reported as 1.02. Confidence interval (CI) is 95%: 0.92–1.13.	[161]
Potassium-competitive acid blockers (P-CABs)	Clinical	The study included 232 treatment-naïve participants divided into two groups: Arm 1 (58 patients) received clarithromycin, amoxicillin, and vonoprazan, while Arm 2 (58 patients) received clarithromycin, amoxicillin, and esomeprazole. Treatment-experienced patients were in Group II, consisting of Arm 3 (intervention) and Arm 4 (comparator), each with 58 participants. Arm 3 received levofloxacin, vonoprazan, nitazoxanide, and doxycycline, while Arm 4 received levofloxacin, esomeprazole, nitazoxanide, and doxycycline. All participants followed their treatment for 14 days, with *H. pylori* eradication assessed four weeks later.	Arm 1 had the highest eradication rate at 58.6%. Arm 2 and 3 recorded a 50% eradication rate. Arm 4 had the lowest eradication rate at 43.1%. Regimens containing P-CABs were well-tolerated by participants. There were few adverse events reported in the study.	[167]
Probiotics	Clinical	This double-blind, randomized controlled trial enrolled 450 patients with *H. pylori* infection. Participants received a 14-day quadruple treatment of bismuth subcitrate, pantoprazole, amoxicillin, and clarithromycin, and were randomly assigned to either a probiotic (*Lactobacillus ruteri*, 100 mg) or a placebo. Eight weeks post-therapy, a urea breath test assessed *H. pylori* eradication rates, the primary outcome, while side effects were evaluated as a secondary outcome.	The probiotic group showed higher *H. pylori* eradication rates compared to the placebo group. Eradication rates were 80.1% for probiotics vs. 75.2% for placebo (per-protocol) and 78.7% vs. 72% (intention-to-treat). Side effects were reported by 69.7% of the probiotic group, significantly lower than 98.6% in the placebo group (*p* < 0.001). The probiotic group experienced fewer gastrointestinal adverse effects, with the exception of constipation (*p* < 0.001).	[174]
	Clinical	The study involved 95 *H. pylori*-positive participants and 56 negative controls, aged 19 to 30, assigned to probiotics monotherapy, probiotics-supplemented quadruple therapy, or quadruple therapy alone. Gastric mucosal samples were collected before treatment and two months later for *16S rRNA* gene sequencing. Two months after eradication, the gastric microbial composition significantly differed from that of *H. pylori*-negative participants, with decreased alpha diversity in gastric juice and increased diversity in gastric mucosa.	Eradicating *H. pylori* from the stomach microbiota in young adults disrupts the microbial balance. Recovery from this disruption takes time. Probiotics can help correct dysbiosis caused by eradication therapy. Young individuals may require additional treatments to effectively combat *H. pylori* infection.	[180]
Lactoferrin therapy	Preclinical in vitro	An investigation was conducted to evaluate the antibacterial properties of lactoferrin and Lactoferricin^®^, an antimicrobial peptide derived from lactoferrin, against *H. pylori*.	Bovine Lactoferricin^®^ demonstrated effectiveness at concentrations above 5.0 mg/L. Human and bovine lactoferrins had minimum bactericidal concentrations ranging from 1.25 to 2.50 mg/mL, indicating a dose-dependent effect. Iron-saturated lactoferrin did not inhibit bacterial growth. Bovine Lactoferricin^®^ was effective in reducing urease activity in *H. pylori*.	[204]
	Preclinical in vivo	The impact of bovine lactoferrin (bLF) on germ-free BALB/c mice infected with *H. pylori* was examined. After oral inoculation with *H. pylori*, the mice were given bLF daily for either two or four weeks. The mice were then euthanized to evaluate serum antibody levels and bacterial counts in the stomach. To isolate *H. pylori* attached to the gastric epithelium, the stomachs were agitated in phosphate-buffered saline.	Administering 10 mg of bLF over three to four weeks resulted in a tenfold increase in *H. pylori* presence in the stomach. There was a significant reduction in *H. pylori*’s attachment to the gastric epithelium. Serum antibody titers for *H. pylori* dropped to undetectable levels, indicating a weakened immune response. The findings suggest that bLF has a direct antibacterial effect. BLF can detach *H. pylori* from the stomach epithelium.	[205]
Phytotherapy	Preclinical in vitro and in vivo	This study examines the effects of Banxia Xiexin Decoction (BXXXT), a traditional Chinese medicine prescription, on drug-resistant *H. pylori*-induced gastritis in mice using in vivo and in vitro methods. The aqueous extract of BXXXT was prepared by water decoction. In vitro tests indicated that BXXXT inhibits *H. pylori*. An acute gastritis model was established in vivo to assess *H. pylori* colonization, gastric mucosal repair, inflammation, and apoptosis in treated mice.	The BXXXT aqueous extract has a minimum inhibitory concentration of 256–512 μg/mL against *H. pylori.* This concentration is higher than the standard triple therapy dosage of 28 mg/kg. The extract contains at least 11 compounds, including berberine and quercetin, which may have synergistic effects. It significantly enhances CD3+ and CD4+ T cell expression in gastritis mice. The extract improves the CD4+/CD8+ T cell ratio in gastric mice. It targets CFAs related to urea enzymes, cagA, and vacA.	[216]
	Preclinical In vitro and in silico	The essential oils and methanol extracts of *Pimenta racemosa* (*P. racemosa*) leaves and stems were studied for their potential inhibitory activities against *H. pylori* both in vitro and in silico. The antibacterial activity of the essential oils and methanol extracts against *H. pylori* was evaluated using the micro-well dilution technique.	Essential oil from stems shows inhibition of *H. pylori* with an MIC value of 3.9 µg/mL. This MIC value is comparable to clarithromycin, which has an MIC value of 1.95 µg/mL. Molecular modeling studies indicate potential inhibitory effects on *H. pylori* urease from compounds such as decanal, eugenol, terpineol, delta-cadinene, and amyl vinyl.	[221]
Phototherapy	Preclinical In vitro	A bacteria-targeted near-infrared (NIR) photosensitizer, designated T780T-Gu, has been developed through the combination of positively charged guanidinium (Gu) and the effective phototherapeutic agent T780T.	T780T-Gu is effective in both photothermal and photodynamic treatments. It targets biofilms and multidrug-resistant (MDR) strains of *H. pylori*. The treatment’s effectiveness may be enhanced by structural deficits and reduced metabolism in the bacteria.	[248]
	Preclinical In vivo	The authors have developed a poly-L-lysine-based photomedicine conjugated with multiple 3SL (p3SLP). They proposed a targeted PDT strategy utilizing an endoscopic laser system for the treatment of *H. pylori*. The antibacterial efficacy of p3SLP was evaluated in C57BL/6 mice infected with *H. pylori*.	P3SLP is administered orally and exposed to laser irradiation. It effectively inactivates *H. pylori* by targeting the sabA protein on the bacterial membrane. The treatment does not harm host mammalian cells. P3SLP shows potential as an endoscopic antibacterial PDT method for treating *H. pylori*.	[228]
Phage therapy	Preclinical In vitro	Prophage isolation using *H. pylori* strains and UV radiation led to the identification of HPy1R, a new podovirus with a genome of 31,162 bp and a GC content of 37.1%. It encodes 36 predicted proteins, 17 of which are structural. The phage remains stable at 37 °C and pH levels from 3 to 11 for 24 h.	HPy1R demonstrated a slight decrease in viability in an in vitro gastric digestion model, indicating adaptation to gastric conditions. It effectively suppressed *H. pylori* populations for up to 24 h after infection. HPy1R is considered a promising candidate for phage therapy, especially in the absence of strictly lytic phages.	[255]
	Preclinical In vitro	The effectiveness of *H. pylori*-specific lytic phage (*H. pylori* φ) alone and with lactoferrin adsorbed on hydroxyapatite (LF-HA) nanoparticles (*H. pylori* φ + LF-HA) in preventing *H. pylori* infection. The bacteria were obtained from human stomach biopsies and cultured in brain heart infusion (BHI) broth with 10% horse serum at 37 °C and 5% CO_2_ for phage isolation.	LF-HA significantly enhances the activity of *H. pylori* phages. Phages complexed with LF can selectively target and eliminate *H. pylori* while preserving host cells. This combination presents a promising therapeutic option for *H. pylori* infections. The *H. pylori* φ + LF-HA combination shows potential efficacy when given at the onset of infection. Minimum effective doses for the treatment have not yet been established.	[256]
Vaccine development	Preclinical in vivo	The multivalent epitope-based vaccine CFAdE was developed from antigenic fragments of four Helicobacter pylori adhesins: urease, *Lpp20*, *HpaA*, and *cagL*. Its specificity, immunogenicity, and ability to generate neutralizing antibodies were tested in BALB/c mice, followed by evaluations in *H. pylori*-infected Mongolian gerbils.	CFAdE induces antibodies against several targets: urease, Lpp20, HpaA, and cagL. Oral vaccination with CFAdE combined with a polysaccharide adjuvant (PA) significantly decreased *H. pylori* colonization. The reduction in colonization was associated with increased levels of IgG, sIgA antibodies, and antigen-specific CD4+ T cells. A multivalent vaccine that targets multiple adhesins demonstrated greater efficacy compared to a vaccine focused solely on urease. The findings suggest the potential of multivalent vaccines in combating *H. pylori* infection.	[270]
	Preclinical In vivo	*Bacillus subtilis* spores were engineered to display potential *H. pylori* protective antigens, urease subunit A (*ureA*), and subunit B (*ureB*), on the spore surface. Immunity and colonization in mice challenged with *H. pylori* after orally administering these spores were tested.	The vaccination reduces *H. pylori* colonization by up to 1 log. The study emphasizes the potential of Bacillus spores for mucosal immunization against *H. pylori*. Bacillus spores are noted for their thermal stability and probiotic properties.	[271]
	Clinical Phase 3 trial)	A phase 3 clinical study in China evaluated a three-dose oral recombinant *H. pylori* vaccine’s effectiveness, safety, and immunogenicity in healthy children aged six to fifteen. Participants without prior infection were randomly assigned to receive the vaccine or a placebo, with the primary outcome being the incidence of infection within one year. Registered with ClinicalTrials.gov (NCT02302170), the trial enrolled 4464 individuals from 2 December 2004, to 19 March 2005, with 4403 (99%) completing the regimen.	A total of 64 *H. pylori* infection cases were reported in the first year. Breakdown of cases: 14 in the vaccination group and 50 in the placebo group. Vaccine effectiveness was calculated at 71.8% with a 95% confidence interval of 48.2–85.6. Adverse reactions occurred in 157 (7%) of the vaccinated group and 161 (7%) of the placebo group. Major adverse events were reported in five (<1%) individuals in the vaccinated group and seven (<1%) in the placebo group. None of the major adverse events were linked to the vaccine.	[272]

## Data Availability

Not applicable.
